# Optimization of Exopolysaccharides Production by *Porphyridium sordidum* and Their Potential to Induce Defense Responses in *Arabidopsis thaliana* against *Fusarium oxysporum*

**DOI:** 10.3390/biom11020282

**Published:** 2021-02-14

**Authors:** Marwa Drira, Jihen Elleuch, Hajer Ben Hlima, Faiez Hentati, Christine Gardarin, Christophe Rihouey, Didier Le Cerf, Philippe Michaud, Slim Abdelkafi, Imen Fendri

**Affiliations:** 1 Laboratoire de Biotechnologies des Plantes Appliquées à l’Amélioration des Cultures, Faculté des Sciences de Sfax, Université de Sfax, 3038 Sfax, Tunisia; maroua.drira.etud@fss.usf.tn; 2 Laboratoire de Génie Enzymatique et Microbiologie, Equipe de Biotechnologie des Algues, Ecole Nationale d’Ingénieurs de Sfax, Université de Sfax, 3038 Sfax, Tunisia; Jihen.elleuch@gmail.com (J.E.); hajer.benhlima@enis.tn (H.B.H.); faizhentati@gmail.com (F.H.); slim.abdelkafi@enis.tn (S.A.); 3 Université Clermont Auvergne, CNRS, SIGMA Clermont, Institut Pascal, F-63000 Clermont-Ferrand, France; chistine.gardarin@uca.fr; 4 Normandie University, UNIROUEN, INSA Rouen, CNRS, PBS, 76000 Rouen, France; christophe.riouey@univ-rouen.fr (C.R.); didier.leerf@univ-rouen.fr (D.L.C.)

**Keywords:** *Porphyridium*, exopolysaccharides, *Fusarium*, *Arabidopsis*, elicitation, salicylic acid pathway

## Abstract

Polysaccharides from marine algae are one novel source of plant defense elicitors for alternative and eco-friendly plant protection against phytopathogens. The effect of exopolysaccharides (EPS) produced by *Porphyridium sordidum* on elicitation of *Arabidopsis thaliana* defense responses against *Fusarium oxysporum* was evaluated. Firstly, in order to enhance EPS production, a Box–Behnken experimental design was carried out to optimize NaCl, NaNO_3_ and MgSO_4_ concentrations in the culture medium of microalgae. A maximum EPS production (2.45 g/L) higher than that of the control (0.7 g/L) was observed for 41.62 g/L NaCl, 0.63 g/L NaNO_3_ and 7.2 g/L MgSO_4_ concentrations. Structurally, the EPS contained mainly galactose, xylose and glucose. Secondly, the elicitor effect of EPS was evaluated by investigating the plant defense-related signaling pathways that include activation of Salicylic or Jasmonic Acid-dependent pathway genes. A solution of 2 mg/mL of EPS has led to the control of fungal growth by the plant. Results showed that EPS foliar application induced phenylalaline ammonia lyase and H_2_O_2_ accumulation. Expression profile analysis of the defense-related genes using qRT-PCR revealed the up-regulation of Superoxide dismutases (SOD), Peroxidase (POD), Pathogenesis-related protein 1 (PR-1) and Cytochrome P450 monooxyge-nase (CYP), while Catalase (CAT) and Plant defensin 1.2 (PDF1.2) were not induced. Results suggest that EPS may induce the elicitation of *A*. *thaliana*’s defense response against *F. oxysporum*, activating the Salicylic Acid pathway.

## 1. Introduction

Plants are usually exposed to a wide range of phytopathogenic microorganisms and viruses and have developed different mechanisms of defense to control their attacks. These mechanisms vary from constitutive to induced defenses. Constitutive defenses are designed by physical barriers such as rigid cell walls and wax epidermal cuticles, while inducing defense responses are activated by a wide variety of elicitors, which are made up of diverse molecular structures such as peptides, lipids and carbohydrates [1]. These products comprise both pathogen- and plant-derived molecules. Pathogen-issued compounds are exogenous elicitors including lipopolysaccharides and flagellin from bacteria and mannoproteins, β-glucans and chitosan from fungi. Plant-derived molecules called endogenous elicitors are notably oligogalacturonides and cutin monomers [2]. Once a microbial or viral attack is perceived, a complex array of defense responses is trigged, leading to the production of reactive oxygen species, phytoalexins and a set of endogenous signal molecules including jasmonic acid (JA), ethylene (ET) and salicylic acid (SA) [3].

In agriculture management, elicitor treatment in the absence of pathogens is an effective strategy to mitigate the negative impact of plant infection on crop yield [4]. Marine algae, as an abundant source of polysaccharides, is one novel source of plant defense elicitors [5,6]. Several understandings suggest that ulvans (from green macroalgae), alginates, fucans and laminarin (from brown macroalgae) and carrageenans (from red macroalgae) induce microbial disease resistance in plants. For example, alginate from *Bifurcaria bifurcate* induced phenylalanine ammonia-lyase (PAL) activity and phenolic compounds accumulation in the leaves of tomato seedlings [7]. Carrageenan from *Hypnea musciformis* elicited expressions of genes involved in SA and JA signaling pathways and contributed to resistance against tobacco mosaic virus infection [8]. Similarly, ulvan and oligoulvans from *Ulva lactuca* induced plant defense response enzymes such as catalase (CAT), superoxide dismutase (SOD), peroxidase (POD) and phenylalanine ammonia-lyase (PAL) and increased lignin and phenolic compounds in apple fruits, which confer resistance against *Penicillium expansum* and *Botrytis cinerea* [9]. More recently, polysaccharides produced by cyanobacteria and microalgae raised serious concern over their role as biostimulants [10]. Nevertheless, only a few studies examined the effect and mode of action of polysaccharides from microalgae as plant protectors. In this context, *Porphyridium sordidum* EPS production was optimized using the Box–Behnken experimental design. The obtained EPS were then structurally characterized. Their elicitor effects on *Arabidopsis thaliana* leaves against *Fusarium oxysporum* infection were evaluated. Finally, *A. thaliana*’s defense reactions to fungal infection were investigated.

## 2. Materials and Methods

### 2.1. Strain Isolation and Growth Conditions

The red microalgae *Porhyridium* sp. was isolated from Mahdia seawater in central-eastern Tunisia (35°30′ N, 11°04′ E) using micromanipulation techniques. The monoalgal stain was cultured in Pm medium [11] containing: 28 g/L of NaCl, 1.7 g/L of NaNO_3_, 0.9 g/L of K_2_HPO_4_, 7.2 g/L of MgSO_4_, 7H_2_O, 1.55 g/L of CaCl_2_, 2H_2_O, 1 mL/L of trace metal solution composed of 2 mg/L H_3_BO_3_, 1.8 mg/L MnCl_2_,4H_2_O 0.02 mg/L (NH4)_6_ Mo_7_ O_24_, 4H_2_O, 213 mg/L ZnSO_4_,7H_2_O, 0.08 mg/L CuSO_4_,5H_2_O and 91 mg/L CoSO_4_ and 1 mL/L of vitamin solution containing 0.5 mg/L of Vitamin B_12_, 100 mg/L of Vitamin B1 and 0.5 mg/L of Biotin. The pH of the medium was adjusted to pH 7.5. The culture was kept at 20 °C and at a luminous intensity of 150 μmol photons/m²/s with continuous agitation 100 rpm. The biomass increasing in the culture medium was followed measuring A_750_.

### 2.2. Genomic DNA Extraction and Molecular Identification of the Isolated Strain

Two mL of biomass at the exponential growth phase was centrifuged at 10,000 rpm for 10 min and used to extract genomic DNA. The pellet was solubilized in 1 mL of the lysis buffer (0.1 M Tris-HCl pH 8, 0.5 M NaCl, 0.05 M EDTA pH 8 and 10% SDS). After incubation for 1 h at 65 °C, 1 mL of chloroform / isoamyl alcohol (24/1) was added and the sample was homogenized by agitation for 30 min. Then, the upper aqueous phase obtained after centrifugation at 3000 rpm for 15 min was precipitated with cold absolute ethanol. The samples were centrifuged at 12,000 rpm for 5 min. The supernatant was then removed, and 1 mL of cold ethanol 70% was added. The mixture was then centrifuged at 12,000 rpm for 5 min and the supernatant was discarded. When fully dried, the pellet was resuspended in 50 μL of ultra-pure water. The 18S rRNA gene sequence of the isolated strain was amplified using the universal primers EukA and EukB as described by Ben Amor et al. [12] and Dammak et al. [13]. After sequencing of the PCR product, 18S rRNA sequences of the isolated strain were compared with the microalgal sequences available in the Gene Bank database using the BLAST program. Then, a phylogenetic tree was constructed using the neighbor joining method to show the phylogenetic relationships of the isolated strain (*Porphyridium sordidum* MD) and the closely related taxa. Bootstrap values are shown on nodes in percentages of 500 replicates for values over 70%. In the substitution model, the maximum composite likelihood method implemented in the Molecular Evolutionary Genetics Analysis version 6.0 (MEGA 6) software was used. *Erythrotrichia carnea* strain SAG 31.94 (AJ880417.1) was presented as an outgroup. The scale bar0.2 represent changes per nucleotide position [14,15].

### 2.3. Extraction of Exopolysaccharides (EPS) from P. sordidum

Ten mL of the culture medium of *P. sordidum* was centrifuged at 10,000 rpm for 10 min at 4 °C. Three volumes of ice-cold absolute ethanol (−20 °C) were added to the supernatant, and the mixture was kept at 4 °C for 12 h. The precipitated EPS were collected after centrifugation at 6000 rpm for 20 min. The pellet was then dissolved in ultra-pure water. The dissolved EPS were dialyzed against distilled water for 2 days in a dialysis tubing cellulose membrane (molecular weight cut off = 10,000 Da). The dialyzed EPS solution was then frozen and freeze dried. The total sugar content of EPS was measured using the phenol-sulfuric acid method using glucose as standard [16]. Sulfate content in EPS was determined by the Barium-gelatin method using K_2_SO_4_ as standard [17,18].

### 2.4. Experimental Design and Data Analysis

The determination of the optimal conditions for maximal EPS production using *P. sordidum* was carried out using an experimental design based on the response surface methodology (RSM). Three independent variables were tested: NaCl (X1), NaNO_3_ (X2) and MgSO_4_ (X3) concentrations. Table 1 shows the coded levels and the experimental range of each variable. A Box–Behnken experimental design involving 15 experiments with the three variables at three levels (low (−1), medium (0), and high (+1)) was employed. EPS concentration (Y) was fixed as the design experiments response, which could be described by a second order polynomial function. The value of the dependent response was the mean of two independent experiments. The independent parameters and the dependent output response were modelled and optimized using analysis of variance (ANOVA) to justify the adequacy of the models using Nemrod-W^®^ software (LPRAI, Marseille, France). The chosen confidence interval is of the order of 95%, corresponding to *p* < 0.05. The second-order polynomial model for predicting optimal conditions can be expressed according to Equation (1).

Y =β_0_ + Σβ_i_X_i_ + Σβ_ij_X_i_X_j_ + Σβ_ii_X_i_^2^ (1)

where Y is the predicted response (EPS concentration); β_0_ is the intercept term; β_i_ is the linear coefficient; β_ii_ is the quadratic coefficient; and β_ij_ is the interaction coefficients. X_i_ and X_j_ are the levels of the independent variables.

The response surface graphs indicate the effect of variables individually and in combination and determine their optimum levels for maximal EPS production. They were performed using Nemrod-W^®^ software (LPRAI, Marseille, France). To validate these predictions, experience using completely optimized conditions was performed in triplicates later.

### 2.5. Structural and Physic-Chemical Characterizations of EPS

#### 2.5.1. HPAEC-PAD Analysis of EPS

Analysis of the EPS by High Performance Anion Exchange Chromatography with Pulsed Amperometric Detection (HPAEC-PAD) required prior hydrolysis of 15 mg of polysaccharides in 1.5 mL of trifluoroacetic acid (TFA) (2 M) for 90 min at 120 °C to release the monosaccharides. The obtained hydrolysate was then neutralized with a 33% NH_3_ solution and centrifuged at 13,000× *g* for 15 min at 4 °C. Different dilutions from a stock concentration of 10 g/L were prepared and filtered through 0.22 μm. Samples (25 µL) were then analyzed by HPAEC-PAD using a Dionex ICS-3000 system with an SP gradient pump, an Ag/AgCl reference electrode, an ED electrochemical detector coupled with a gold performance electrode, and a Chromeleon version 6.5 (Dionex Corp., Bannockburn, IL, USA). It was assembled with a guard CarboPac PA1-column (4 × 50 mm) and analytical CarboPac PA1-column (4 × 250 mm).

Before each injection, columns were equilibrated by running for 15 min with 18 mM NaOH. Samples were eluted isocratically with 18 mM NaOH for 25 min, followed by a linear gradient between 0 to 0.5 M sodium acetate in 200 mM NaOH for 20 min to elute acidic monosaccharides. Run was followed by 15 min of washing with 200 mM NaOH. The eluent flow rate was kept constant at 1 mL/min. Columns were thermostated at 25 °C.

Samples were injected in a complete loop of 25 μL at a concentration of 1 mg/mL.

#### 2.5.2. HPSEC-MALLS Analysis of EPS

The determination of the macromolecular magnitudes (i.e., the mass average molar mass (M_w_), number average molar mass (Mn), gyration radius (Rg), hydrodynamic radius (Rh) and intrinsic viscosity ([η]) of EPS in the diluted regime was carried using high pressure size exclusion chromatography (HPSEC) [18]. The HPSEC-MALLS was equipped with three detectors online: a multi-angle laser light scattering (MALLS) filled with a He–Ne laser at 690 nm and a K5 cell (50 μL) (HELEOSII, Wyatt Technology, Goleta, CA, USA), a differential refractive index (DRI) (RID10 A Shimadzu, Japan) as well as a viscosimeter (Viscostar II, Wyatt Technology, CA, USA). Columns (OHPAK SB-G guard column, OHPAKSB806 and 804 HQ columns (Shodex, Showa Denko, Japan)) were eluted with LiNO_3_ (0.1 M) at a flow rate of 0.7 mL/min. Note that the solvent was filtered through a 0.1 μm filter unit (Millipore, Burlington, MA, USA), degassed and filtered using a 0.45 μm filter upstream column. EPS was solubilized at a concentration of 2 g/L in 0.1 M of LiNO_3_ solution for 24 h under stirring, filtered (0.45 μm), and then injected through a 500 μL full loop. The collected analyses data were analyzed involving a Astra 6.1 software package (Wyatt technology, CA, USA) and a dn/dc of 0.15 mL/g.

### 2.6. Plant and Fungal Growth Conditions

Seeds of wild-type *A. thaliana* (ecotype Columbia) and were surface-sterilized, plated on Murashige and Skoog (MS) agar medium [19], and incubated 2 days at 4 °C in darkness conditions. Then, the plates were transferred to the growth chamber at 22 °C with a light intensity of 250 µmol/m^2^/s under long-day conditions 16 h/8 h light/dark cycle. After one week, the seedlings were transplanted to peat in plastic pots. Pots were placed on a phytotron under the same conditions. Four-week-old plants were used in the experiments.

*F. oxysporum* (CTM10402) was grown on autoclaved potato dextrose agar (PDA) plates at 25 °C for 48 h in the dark. Spores were collected and adjusted to 10^6^ spores/mL for plant infestation.

### 2.7. Elicitor Treatment and Infestation

Treatments were applied to the *A. thaliana* leaves twice before infestation. Plants with fully expanded leaves were sprayed with different concentrations of EPS (1, 1.5 or 2 mg/mL) solutions, followed by a second spray treatment after 24 h. The control plants were sprayed with sterile distilled water. One day later, leaves were punctured with a needle, and 5 μL of the spore suspension (one drop) was placed over the wound. Infected plants were then covered with dark plastic bags for 48 h. Leaves were harvested, photographed and then frozen at −80 °C for further analysis.

### 2.8. Determination of Hydrogen Peroxide (H_2_O_2_) Content

Samples of fresh leaves (100 mg) were homogenized with 1 mL of trichloroacetic acid (TCA) at 0.1% (*w*/*v*) and centrifuged at 10,000 rpm for 15 min at room temperature. H_2_O_2_ content was determined following the method of Velikova et al. [20]. Briefly, one mL of supernatant was added to 1 mL of 10 mM phosphate buffer (pH 7) and 2 mL of a KI solution (1 M). Finally, the A_390_ of the mixture was measured, and the H_2_O_2_ content was calculated using H_2_O_2_ solutions ranging from 0.1 to 1 mM as standard.

### 2.9. Extraction and Measurement of Phenylalanine Ammonia Lyase (PAL) Activity

Leaf samples (150 mg fresh weight) were extracted in 2 mL of buffer (100 mM Tris pH 8.8, 14 mM 2-mercaptoethanol and 3% *w*/*v* polyvinylpolypyrrolidone) and centrifuged at 10,000 rpm for 10 min at 4 °C. The supernatant was collected, and the total protein concentration was determined using the Bradford assay [21]. PAL activity was measured according to the procedure described by El-Shora et al. [22]. The reaction mixture contained 1.9 mL of 100 mM Tris–HCl buffer (pH 8.8), 1 mL of 15 mM l-phenylalanine and 100 µL of enzyme extract. After incubating at 30 °C for 15 min, the reaction was stopped by adding 200 µL of 6 M HCl, and A_290_ was measured. One unit of PAL activity was defined as the number of enzymes leading to the conversion of 1 mmol of l-phenylalanine into trans-cinnamic acid per minute. The trans-cinnamic acid synthesized was calculated using its molar extinction coefficient (9630/M/cm) and expressed as nmol trans-cinnamic acid/min/mg of protein.

### 2.10. Extraction of RNA, Synthesis of cDNA and Real-Time Quantitative PCR

Extraction of total RNA was performed using the leaves of *A. thaliana* with the plant-RNA mini kit (Qiagen, Germantown, MD, USA) in accordance with the manufacturer’s instruction. RNA concentrations and purity were determined by measuring A_260_/A_280_ ratios using a NanoPhotometer™ (Implen, GmBH, Konstanz, Germany). After the DNase-treatment of RNA samples, 5 µg of total RNA was used to synthesize cDNA using M-MLV reverse transcriptase (Invitrogen, Thermo Fisher Scientific, Waltham, MA, USA). Real-Time qPCR was conducted for six defense-related genes.

These genes were encoded as follows: SOD, CAT, POD, PR1, CYP and PDF1.2 (Table 2). Amplification reactions were performed in 15 μL final volumes containing 7.5 μL of 2X SYBR Remix Ex Taq II (Takara, Kyoto, Japan), 1.5 μL of primer-pair mix (0.5/0.5 μM for forward and reverse primers), 3 μL of cDNA, and 3 μL of RNase-free water. Reactions were carried out in a onStepOnePlus™ PCR cycler (Applied Biosystems, Foster City, CA, USA). Thermal cycling conditions were 5 min at 95 °C, followed by 45 cycles of 20 s at 95 °C, 15 s at 60 °C, and 20 s at 72 °C. Routinely, the specificity of the primers was confirmed by a melting curve performed at the end of the qPCR cycles. Data were analyzed from two independent Real-Time PCR runs. Transcript abundance of each selected gene was normalized to the housekeeping gene actin (ACT1) and Cytoplasmic Glyceraldehyde-3-Phosphate Dehydrogenase (GAPC2) as housekeeping genes and was determined with the delta delta Ct (ΔΔCt) method [23].

## 3. Results

### 3.1. Isolation and Identification of the New Porphyridium Strain

**Figure 1:**
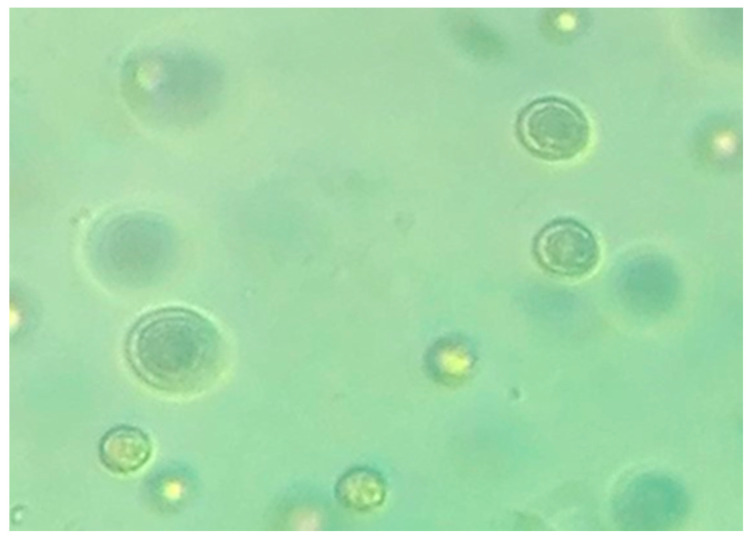
Microscopic observation (at 40× magnification) of the microalgae strain *P. sordidum.*

The new marine microalgae isolated from the Tunisian seawater was preliminarily identified as being of the genus *Porphyridium* sp. because it presented cells with diameter between 7–10 μm, had a spheric shape, was devoid of flagella and represented an olive-green color (Figure 1). In fact, this color is characteristic of the species *P. sordidum*, which is considered as valid taxon distinct from other members of the genus *Porphyridium*, namely the blood-red *P. cruentum* and *P. purpureum* [24,25].

**Figure 2:**
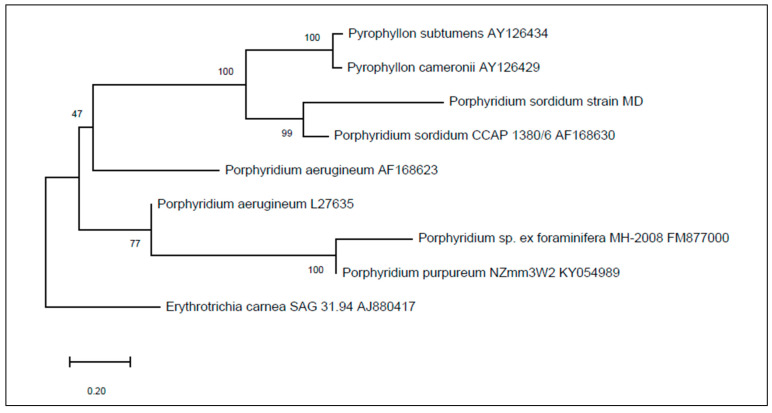
Phylogenetic trees derived from the 18S rDNA sequence of the isolated strain. Genbank accession numbers for all species are given to the right of each branch. Numbers at nodes indicate levels of bootstrap support based on a neighbour-joining analysis of 500 resampled datasets. Bar—0.020 substitutions per nucleotide position.

Although morphological analysis is an important method for the identification of microorganisms, molecular analyses is required to confirm their identity. On the basis of the NCBI (National Center for Biotechnology Information) blast results of the 18S rRNA gene sequence and phylogenetic analysis (high bootstrap value) (Figure 2), the isolated strain belonged to the genus *Porphyridium* and was closely related with *P. sordidum*.

### 3.2. Kinetics of Growth and EPS Production of P. sordidum under Standard Conditions

**Figure 3:**
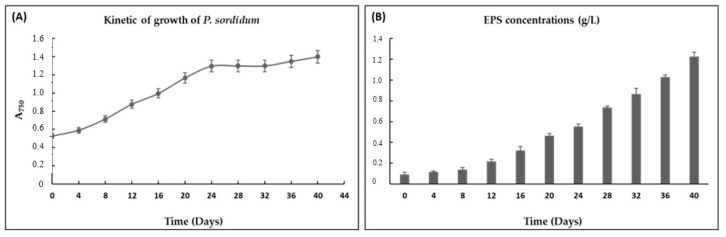
Kinetics of growth (**A**) and exopolysaccharides (EPS) (**B**) production of *P. sordidum* under standards conditions.

The culture medium was studied at 20 °C using Pm medium under a luminous intensity of 150 μmol photons/m²/s and continuous agitation of 100 rpm. Growth of microalgae was followed by spectrophotometry (A_750_) for 40 days. As shown in Figure 3A, *P. sordidum* behaved like a slow-growing species with a growth rate (µ) of 0.055 day^−1^ and a doubling time (Td) of 12.6 days. This growth behavior was described recently by Medina-Cabrera et al. [25], showing that, like diatoms, *P. sordidum* with large cell size (~10 µm) had a lower growth rate compared with the small cells of *P. purpureum*. The exponential phase began on day 4 and ended on day 24.

Figure 3B gives insight into the EPS synthesis that occurred along the growth phase. EPS production was observed during all growth phases but remarkably increased during the end of the exponential phase and in the stationary phase to reach more than 1 g/L at 40 days. In general, the stationary phase, caused by depletion of different nutrients such as nitrogen and phosphorus, has been reported to induce EPS production of several microalgae such as *Chlamydomonas mexicana* [26] and *Botryococcus braunii* [27].

### 3.3. Structural Characterization of EPS

As shown in Table 3, monosaccharide composition analysis of the isolated EPS led to the identification of a glucoxylogalactan mainly composed of galactose, xylose and glucose, while other sugars such as fucose, glucuronic acid and rhamnose were detected as minor components. These results fit pretty well with those of *P. cruentum*, which were for galactose, xylose and glucose in relative percentages of ~40, 30 and 20, respectively [28]. They are also in agreement with published data focusing on EPS from red microalgae, which are generally identified as galactoxylan or xylogalactan [29].

The Molecular weight distribution of EPS was determined using SEC-MALLS analysis. The weight–average molecular mass (M_w_), number–average molecular mass (M_n_), gyration radius (R_g_), and hydrodynamic radius (R_h_) of EPS extracted from *P. sordidum* were calculated at 14 × 10^5^ g/mol, 12 × 10^5^ g/mol, 120 nm and 66 nm, respectively. According to the literature, the molecular mass of EPS from the *Porphyridium* algal species can be between 0.75 and 2.7 × 10^6^ g/mol. The M_w_ of EPS from *P. sordidum* was in accordance with those of other members of *Porphyridiaceae* (M_w_ of EPS from *P. cruentum* was around 2–7 × 10^6^ g/mol) [30]. As can be seen, the sulfate content (6.7% *w*/*w*) was similar to the ones previously reported in the literature for *Porphyridium* sp., which are generally around 1–9% *w*/*w* [31].

The Intrinsic viscosity of EPS was determined at 1360 mL/g, and the polydispersity index (Đ or Ip) was measured at 1.11, indicating the homogeneity of the EPS extracted from *P. sordidum* (Table 4).

The R_g_/R_h_ ratio can also be used to describe the polysaccharide conformation in solution, sphere conformation (R_g_/R_h_ = 0.77), flexible linear chains (R_g_/R_h_ = 0.82) and stiff rods (R_g_/R_h_ > 2). In this study, it was found at the value of 1.82~2, showing that the EPS isolated from *P. sordidum* presented stiff rods molecular conformation in solution.

The critical overlap concentration of the entanglement of polymer coils (C*) indicates whether a polymer solution is dilute or semi-dilute. It can be defined by the following equation (C* = 4/[η]), represented as the limit between the dilute and semi-dilute regimes. The theoretical C* value of EPS in water at 25 °C was calculated from Table 4 and is about 2.94 g/L.

### 3.4. Optimization of EPS Production by P. sordidum

It is well established that EPS production is affected by environmental constraints and nutrient limitations. In fact, a large number of studies support that N and S are crucial nutrients for microalgae growth; however, their limitation in the culture medium may promote EPS production. Furthermore, hypersalinity is an important physicochemical factor that could enhance EPS production, which protects cells from dehydration [32]. In this study, a response-surface methodology using the Box–Behnken design was applied to determine the optimal levels of three tested variables (NaCl, NaNO_3_ and MgSO_4_ concentrations). The experiment design matrix and the output response are presented in Table 5.

The obtained EPS concentration (Y) results were regressed by a second-order polynomial as a function of the different factors (Equation (2)):

Y = 0.757 + 0.446 X1 − 0.222 X2 − 0.106 X3 + 0.464 X1^2^ + 0.147 X2^2^ − 0.076 X3^2^ − 0.192 X1X2 − 0.075 X1X3 − 0.087 X2X3 (2)

where Y is the predicted EPS concentration, and X1, X2 and X3 are NaCl, NaNO_3_ and MgSO_4_ concentrations, respectively.

The determination coefficient and the adjusted determination coefficient were 95.1% and 86.3%, respectively, indicating that the accuracy and general ability of the polynomial model were acceptable.

The statistical significance of Equation (2) was evaluated by performing ANOVA using the Nemrod-W^®^ software (LPRAI, Marseille, France) software package. As presented in Table 6, the non-significance of the lack of fit indicates that the determined model has a very high quality of fit and is valid in the tested experimental range.

The obtained *p*-values of the quadratic terms of all factors suggested very good dependence of the experimental data on the tested factors (Table 7).

**Figure 4:**
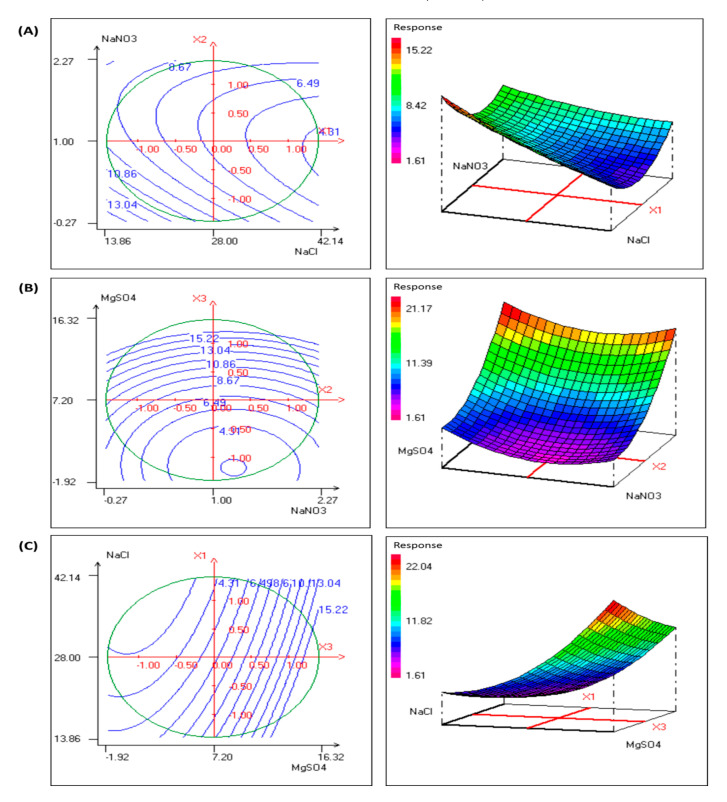
(**A**) Contour plots and response surface plots showing the effect of NaCl and NaNO_3_ concentrations and their mutual interaction on fixed MgSO_4_ concentration. (**B**) Contour plots and response surface plots showing the effect of NaNO_3_ and MgSO_4_ concentrations and their mutual interaction on fixed NaCl concentration. (**C**) Contour plots and response surface plot showing the effect of MgSO_4_ and NaCl concentrations and their mutual interaction on fixed NaNO_3_ concentration.

The relationships between EPS concentration and experimental levels of each tested variable were presented in 2D and 3D response surface plots (Figure 4). The surface plots analysis showed significant interactions between each pair of variables, describing the ANOVA results obtained in this work (Table 7).

The optimum values of the tested variables were obtained by solving Equation (2) and also analyzing the response surface plots. Maximum EPS concentration could be achieved when NaCl, NaNO_3_ and MgSO_4_ concentrations were set to 41.62 g/L, 0.63 g/L and 7.2 g/L, respectively. Using these suggested conditions, the EPS concentration was 2.42 g/L, which was in agreement with the predicted value (2.45 g/L). At the end of the experiment under completely optimized conditions, sulfate content in EPS was determined and expressed as a percentage of the total amount of EPS. It was about 8.21%. It is worth noting that this marginal increase in sulfate content compared with standard conditions (6.7%) may be the result of protein (such as phycoerythrin) degradation under nitrogen limitation [33].

### 3.5. Effect of EPS Pretreatments on Subsequent F. oxysporum Infection in A. thaliana Plants

**Figure 5:**
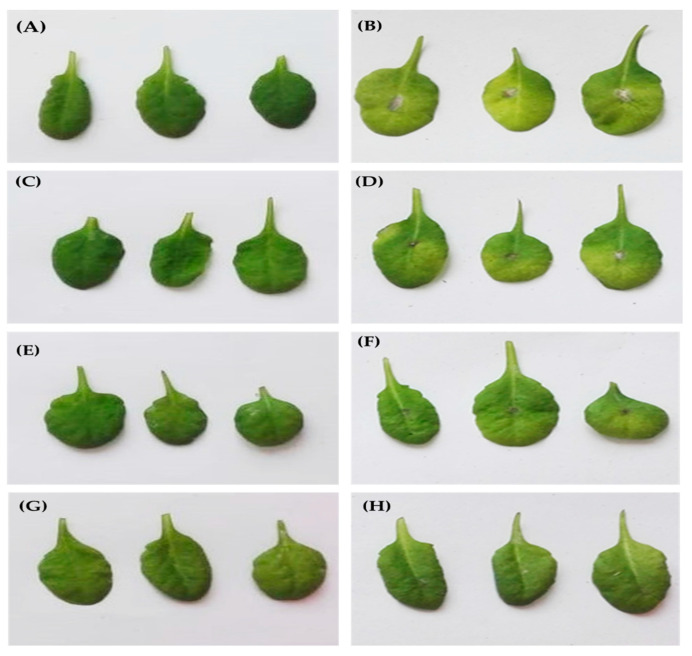
The manifestation of disease symptoms on EPS-treated *A. thaliana* leaves after 48 h of *F.* *oxysporum* infection. (**A**) Untreated control leaves; (**B**) untreated leaves inoculated with *F. oxysporum*; (**C**) leaves pretreated with 1 mg/mL of EPS; (**D**) leaves pretreated with 1 mg/mL of EPS and inoculated with *F. oxysporum*; (**E**) leaves pretreated with 1.5 mg/mL of EPS; (**F**) leaves pretreated with 1.5 mg/mL of EPS and inoculated with *F. oxysporum*; (**G**) leaves pretreated with 2 mg/mL of EPS; (**H**) leaves pretreated with 2 mg/mL of EPS and inoculated with *F. oxyspurum*.

*F. oxysporum* is a fungal pathogen that causes major yield and economic losses and diminishes the crop quality of a broad range of plant species [34]. As mentioned above, elicitor treatment is an effective management practice that may help to control and prevent fungal spread and progress. This finding prompted us to assess the ability of EPS pretreatment to mitigate the injury caused by *F. oxysporum* on the model plant *A. thaliana.* For this reason, leaves were pretreated by spraying different EPS concentrations (1, 1.5 or 2 mg/mL) while control plants were sprayed with water, followed by *F. oxysporum* infection. As shown in Figure 5, after 48 h of inoculation, the leaves from the control plants showed spreading lesions around the site of contact and were surrounded by a yellowish region. However, EPS-treated leaves showed smaller and more restricted lesions around the site of infection (Figure 5D,F,H). Remarkably, the EPS pretreated *A. thaliana* leaves showed a dose-dependent decrease in necrotic lesion development and disease severity after *F. oxysporum* infection. Two mg/mL concentration of EPS seemed the better dose, conferring the most significant resistance against fungal disease (Figure 5H). Such data confirmed the application of EPS as an elicitor that could enhance resistance against *F. oxysporum* infection in plants.

### 3.6. Effect of EPS Treatment on H_2_O_2_ Accumulation and PAL Activity

**Figure 6:**
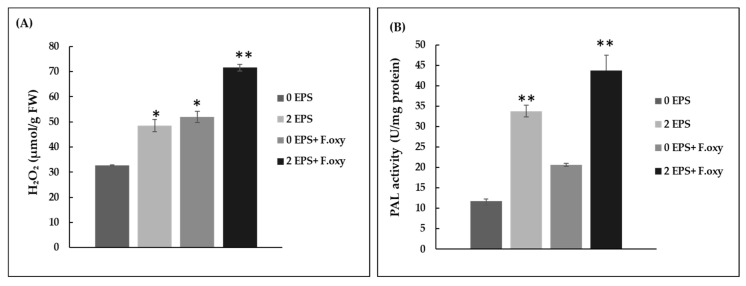
Effects of EPS treatments on H_2_O_2_ accumulation (**A**) and PAL activity (**B**). 0 EPS: leaves sprayed with water; 2 EPS: leaves sprayed with 2 mg/mL of EPS; 0 EPS + *F.* *oxysporum*: leaves sprayed with water and infected with *F.* *oxysporum*; 2 EPS + *F. oxysporum*: leaves sprayed with 2 mg/mL of EPS and infected with *F. oxysporum.* Data are presented as means and standard errors of three repetitions. * indicates significant differences (*p* < 0.05) and ** indicates very significant differences (*p* < 0.01) relative to control untreated plants (Student’s *t*-test).

H_2_O_2_ accumulation and PAL activation were proposed to orchestrate the establishment of plant resistance activation. H_2_O_2_ is an important signal molecule that mediates various defense gene responses [35], and PAL is a key enzyme that plays a crucial role in the phenol synthesis pathway, which is considered the primary inducible response in plants against biotic stresses [36]. Here, it is clear that *A. thaliana* leaves pretreated with a solution of EPS at a concentration of 2 mg/mL showed higher H_2_O_2_ accumulation than those treated with water. Furthermore, the greatest H_2_O_2_ amounts were observed in EPS-pretreated leaves that were infected with *F. oxysporum*, when compared with control inoculated leaves. (Figure 6A). Similarly, PAL activity was found to increase sharply after EPS treatments with or without subsequent *F. oxysporum* infection. Notably, elicitor treatments followed by pathogen infection resulted in a 4- and a 3-fold increase in PAL activity when compared with control and only *F. oxysporum* infected leaves (Figure 6B). These data strongly suggest that EPS have the ability to increase the levels of these biochemical markers of defensive responses like other polysaccharide elicitors [37].

### 3.7. Effect of EPS Pretreatment on the Induction of Plant Defense Gene Expression

**Figure 7:**
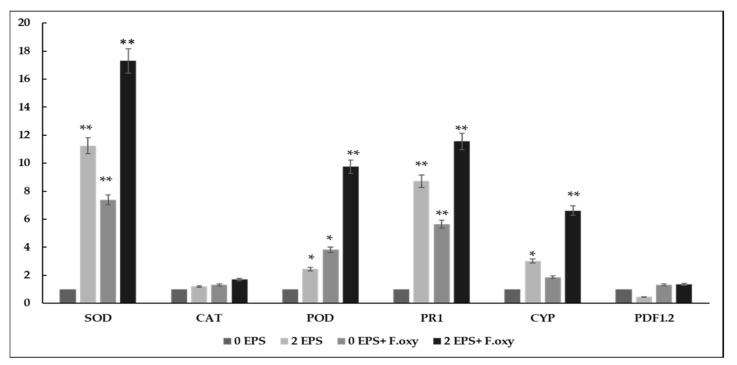
Relative expression levels of defense-related genes SOD, CAT, POD, PR1, CYP and PDF1.2 in *A. thaliana* leaves treated or not treated with 2 mg/mL EPS and infected or not infected with *F. oxysporum.* Transcript abundance of each selected gene is expressed relative to its expression in control, untreated plants. Data were analyzed from two independent Real-Time PCR runs. * indicates significant differences (*p* < 0.05) and ** indicates very significant differences (*p* < 0.01) relative to control untreated plants (Student’s *t*-test).

To better understand the molecular mechanisms underlying EPS-induced *A. thaliana* resistance to *F. oxysporum,* marker genes commonly known to be involved in plants’ defense systems were evaluated. The expression patterns of SOD, CAT and POD (antioxidant gene markers), PR1 (a Salicylic Acid pathway marker), PDF1.2 (a Jasmonic Acid pathway marker) and CYP (belonging to the phytoalexin biosynthesis pathway) were analyzed in EPS-treated and infected plants. As observed in Figure 7, the expression levels of SOD, POD, PR1 and CYP were significantly upregulated by 11-, 2.5-, 9- and 3-fold, respectively, in EPS-treated plants compared with control, untreated plants. A similar trend was also observed in EPS-treated and *F. oxysporum*-infected plants with a 17-, 9-, 1- and 6-fold increase, compared with expression in only pathogen-infected leaves. However, expression levels of CAT and PDF1.2 were found to be the same or lower than basal in EPS-treated plants compared with the controls.

## 4. Discussion

Thanks to their physical and chemical proprieties including high viscosity, high molecular weight, monosaccharides composition, flexibility of the macromolecular chain and sulfation level, microalgae exopolysaccharides have received extensive attention in the last decades [38]. However, despite the multifunctionality of microalgae EPS, their exploration as valuable products is still limited due to their relatively low yield production compared with other microorganisms [39]. In this regard, much research has been focused on the development of optimized strategies for EPS yield improvement. It is well established that EPS are formed in response to adverse conditions such as nutrient limitation and culture conditions (e.g., temperature, agitation, Aeration and light) [32]. For this reason, in the first part of this study the effect of salinity, nitrogen and sulfate on EPS (glucoxylogalactan) production by *P. sordidum* was evaluated using Box–Behnken methodology. Maximum EPS productivity was observed when *P. sordidum* was cultured in a medium characterized by high salinity concentration and nitrogen limitation.

Similar results have been reported by Ozturk et al. [40], Jindal et al. [41] and Borah et al. [42] suggesting that the amounts of EPS produced by different cyanobacteria are increased by the increase of salt concentration in the culture medium and that EPS may protect membranes from desiccation caused by hypersalinity. The same phenomenon was observed by Mishra and Jha., [43] who demonstrated that extracellular salt stress significatively enhanced EPS production by *D. salina.*

As generally acknowledged [32] and as can be ascertained from our results, nitrogen limitation leads to higher EPS production. Li et al. [44] found that *P. purpureum* exhibited the highest EPS content when cultured at low initial nitrogen concentrations (3.5 mM). Silva et al. [45] reported that the best EPS yield in *A. platensis* was achieved under the lowest NaNO_3_ concentration (0.25 g L^−1^). Additionally, Cepák and Přibyl [46] demonstrated that the most abundant EPS levels in *B. braunii* were produced at a moderate nitrogen concentration of 6 mM.

The application of microalgae polysaccharides as bio-elicitors for biotechnological and agronomic strategies to reduce chemical pesticides or fungicides utilization is of great scientific interest. In the present study, EPS’s capacity to induce resistance factors in an *A. thaliana* plant model against *F. oxysporum* was proved. In fact, the application of EPS foliar sprays (2 mg/mL) as a pretreatment remarkably reduced the severity of *F. oxysporum*. These results concur in some previous studies, demonstrating the ability seaweed polysaccharides such alginates, fucans, carrageenans, and ulvans have to induce resistance of plants against pathogens. As examples, laminarin applied to detached grapevine leaves reduced the severity of *B. cinerea* and *P. viticola* [47], alginate was shown to induce resistance against *A. solani* in tomato plants [48], and carrageenan conferred protection of *A. thaliana* against *S. sclerotiorum* [49].

Most interestingly, and to our knowledge, very little light has been shed on the exact mode of action of microalgae polysaccharides as elicitors. It is worth noting that the ability of EPS pretreatments to decrease pathogen disease severity was correlated with the priming of H_2_O_2_ production, PAL activity and some defense genes involved in the antioxidant system, phytoalexin synthesis and the salicylic acid pathway. As it turns out, the early signaling events induced by other elicitors (e.g., chitosan, glucans and peptidoglycans) are firstly designed by a transient accumulation of H_2_O_2_. This key signal mediates plant defense systems by strengthening the antioxidant system by, for example, inducing SOD and POD activities and the accumulation of antimicrobial compounds such as phytoalexins and pathogenesis-related proteins, which altogether play a key role in pathogen restriction [50]. Similar results were reported by Gunupuru et al. [51], suggesting that polysaccharides extracted from *A. nodosum* alone or combined with chitosan had the ability to reduce *Fusarium* head blight in wheat, which also showed an elevated expression in pathogenesis-related protein 1 and β-1,3-glucanase genes and an increase in phenylalanine ammonia lyase, Polyphenol oxidase and Peroxidase enzyme activity. In addition, Jayaraj et al. [52] reported that carrots treated with *A. nodosum* extract reduced the severity of *Alternaria* and *Botrytis* foliar blight disease incidence, which correlated with an enhanced expression of pathogenesis-related protein 1, phenylalanine ammonia lyase, and pathogenesis-related protein 5.

Only one similar approach was also previously performed using polysaccharides from microalgae. Indeed, Rachidi et al. [53] showed that exopolysaccharides from *D. salina* and polysaccharides extracted from *C. vulgaris*, *C. sorokiniana*, and *C. reinhardtii* triggered the innate immunity system of tomato plants depending on the microalgae strain. Higher effects on PAL, β-1,3-glucanase and antioxidant enzymes activities were observed using *C. sorokiniana* and *C. reinhardtii* polysaccharides, and this bioactivity was explained due to their higher uronic acid and sulfate content. Likewise, several reports suggested that effectiveness of polysaccharides as an elicitor was correlated with their chemical structure and believed this to be due to sulfate groups and the degree of sulfation on the repeating galactose and glucose units [54]. More recently, the same authors [55] demonstrated that polysaccharides (PSs) obtained from microalgae and cyanobacteria are considered promising elicitors that are able to induce defense responses in tomato plants. In fact, compared with the laminarin used as control, those PSs have the greatest significant effect on chitinase, peroxidase, glucanase and PAL activities, as well as H_2_O_2_ production.

## 5. Conclusions

Optimal conditions for maximum exopolysaccharides (EPS) production by *P. sordidum* (about 3.4 times higher than that of the control) was attained with concentrations of 41.62 g/L for NaCl, 0.63 g/L for NaNO_3_ and 7.2 g/L for MgSO_4_ in the Pm medium. The first evidence that this sulfated glucoxylogalactan (M_w_ = 14 × 10^5^ g/mol) foliar spray induced resistance to *F. oxysporum* in *A. thaliana* was at an optimal pretreatment concentration of 2 mg/mL. Furthermore, this study demonstrated that EPS are potent elicitors leading to a wide array of defense responses including production of H_2_O_2_, inducing of PAL activity and upregulation of defense-related marker genes such as SOD, POD, CYP and PR1. Overall, these results indicate that EPS may be considered as a novel biological elicitor and an alternative product to chemical fungicides.

## Figures and Tables

**Table 1:** Variables and experimental levels of culture optimization.

Factors	Symbol	Levels		
		−1	0	+1
NaCl (g/L)	X1	18	28	38
NaNO₃ (g/L)	X2	0.1	1	1.9
MgSO₄ (g/L)	X3	0.75	7.2	13.68

**Table 2:** Specific primers used for qRT-PCR.

Gene	Description	Primer (F = forward; R = reverse)
**SOD**	Superoxide dismutase	F 5′-CGCATGATCCTTTGGCTTCG-3′ R 5′-TCCTGGTTGGCTGTGGTTTC-3′
**CAT**	Catalase	F 5′-TCCTGTTATCGTTCGTTTCTCA-3′ R 5′-CAAAGTTCCCCTCTCTGGTGTA-3′
**POD**	Peroxidase	F 5′-CCAAACTCTTCGTGGACTATGC-3′ R 5′-AACTCTTGGTCGCTCTGGAT-3′
**PR1**	Pathogenesis-related protein 1	F 5’-ACGGGGAAAACTTAGCCTGG-3’ R 5’-GCCTTCTCGCTAACCCACAT-3’
**PDF1.2**	Plant defensin 1.2	F 5’- TGCTGGGAAGACATAGTTGC-3’ R 5’- TGGTGGAAGCACAGAAGTTG-3’
**CYP**	Cytochrome P450 monooxygenase	F 5’-GCTGGACCAAATGGGGATCA-3’ R 5’-TCGGCAAACATCGAGACCAA-3’
**ACT**	Actin	F 5’-CATCAGGAAGGACTTGTACGG-3’ R 5’-GATGGACCTGACTCGTCATAC-3’
**GAPC2**	Glyceraldehyde-3-phosphate dehydrogenase C2	F 5’-AGAATTGGACGTTTGGTTG-3′ R 5’-ACTTTGACAGGCTTCTCAC-3′

**Table 3:** Monosaccharides composition (Molar %) of EPS.

Monosaccharides	Fuc	Rha	Ara	Gal	Glc	Xyl	GlcA
Molar%	1.93	0.36	0.36	48.28	19.01	28.2	0.76

**Table 4:** Macromolecular characteristics of EPS.

SulfateContent (*w*/*w*)	Macromolecular Parameters Determined by HPSEC-MALLS						
**6.7%**	**M_n_ (g/mol)**	**M_w_ (g/mol)**	**Đ**	**R_g_ (nm)**	**R_h_ (nm)**	**[η] (mL/g)**	C* (g/L)
	12 × 10⁵	14 × 10⁵	1.1	120	66	1360	2.94

M_w_: weight average molecular mass, M_n_: number average molecular mass, Đ: polydispersity index, R_g_: gyration radius, R_h_: hydrodynamic radius, [η]: intrinsic viscosity and C*: critical overlap concentration. Analyses were run in triplicate, and the relative standard deviations were less than 5%.

**Table 5:** Box–Behnken design of three factors and three levels and the experimental results.

Runing Order	Factors			EPS Concentration (g/L)	
	X1	X2	X3	Y exp	Y pre
1	−1	−1	0	0.93	0.95
2	1	−1	0	2.42	2.23
3	−1	1	0	0.7	0.89
4	1	1	0	1.42	1.40
5	−1	0	−1	0.88	0.73
6	1	0	−1	1.71	1.77
7	−1	0	1	0.73	0.67
8	1	0	1	1.26	1.41
9	0	−1	−1	0.94	1.07
10	0	1	−1	0.84	0.80
11	0	−1	1	0.99	1.03
12	0	1	1	0.54	0.41
13	0	0	0	0.76	0.76
14	0	0	0	0.74	0.76
15	0	0	0	0.77	0.76

**Table 6:** Variance analysis for EPS concentration response.

Source of Variation	Sum of Squares	Degrees of Freedom	Mean Square	Ratio	Significance *
Regression	0.31753	9	0.3528	1512.0706	Significant
Residual	0.1639	5	0.0328		
Lack of fit	0.1639	3	0.0545	233.4643	Not significant
Error	0.0005	2	0.0002		
Total	3.3392	14			

* Statistically significant at 95% of confidence level.

**Table 7:** Coefficient statistical analysis for cell abundance response.

Coefficients	Coefficient Values	Student Test	*p*-Value
b_0_	0.757	85.80	<0.0001
b_1_	0.446	82.63	<0.0001
b_2_	−0.222	−41.20	<0.0001
b_3_	−0.106	−19.67	<0.001
b_11_	0.464	58.39	<0.0001
b_22_	0.147	18.45	<0.001
b_33_	−0.076	−9.54	<0.001
b_12_	−0.192	−25.20	<0.0001
b_13_	−0.075	−9.82	<0.001
b_23_	−0.087	−11.46	<0.001

## References

[1] Hamuel J.D. (2015). An overview of plant immunity. J. Plant Pathol. Microbiol..

[2] Wiesel L., Newton A.C., Elliott I., Booty D., Gilroy E.M., Birch P.R.J., Hein I. (2014). Molecular effects of resistance elicitors from biological origin and their potential for crop protection. Front. Plant Sci..

[3] Mishra A.K., Sharma K., Misra R.S. (2012). Elicitor recognition, signal transduction and induced resistance in plants. J. Plant Interact..

[4] García-Mier L., Guevara-González R.G., Mondragón-Olguín V.M., Verduzco-Cuellar B.D.R., Torres-Pacheco I. (2013). Agriculture and bioactives: Achieving both crop yield and phytochemicals. Int. J. Mol. Sci..

[5] Stadnik M.J., de Freitas M.B. (2014). Algal polysaccharides as source of plant resistance inducers. Trop. Plant Pathol..

[6] Vera J., Castro J., Gonzalez A., Moenne A. (2011). Seaweed polysaccharides and derived oligosaccharides stimulate defense responses and protection against pathogens in plants. Mar. Drugs.

[7] Aitouguinane M., Bouissil S., Mouhoub A., Rchid H., Fendri I., Abdelkafi S., Ould El-Hadj M.D., Boual Z., Dubessay P., Gardarin C. (2020). Induction of natural defenses in tomato seedlings by using alginate and oligoalginates derivatives extracted from Moroccan brown algae. Mar. Drugs.

[8] Ghannam A., Abbas A., Alek H., Al-Waari Z., Al-Ktaifani M. (2013). Enhancement of local plant immunity against tobacco mosaic virus infection after treatment with sulphated-carrageenan from red alga (*Hypnea musciformis*). Physiol. Mol. Plant Pathol..

[9] Abouraïcha E., El Alaoui-Talibi Z., El Boutachfaiti R., Petit E., Courtois B., Courtois J., El Modafar C. (2015). Induction of natural defense and protection against *Penicillium expansum* and *Botrytis cinerea* in apple fruit in response to bioelicitors isolated from green algae. Sci. Hortic..

[10] Chanda M.J., Merghoub N., Arroussi H.E. (2019). Microalgae polysaccharides: The new sustainable bioactive products for the development of plant bio-stimulants?. World J. Microbiol. Biotechnol..

[11] Ben Hlima H., Dammak M., Karkouch N., Hentati F., Laroche C., Michaud P., Fendri I., Abdelkafi S. (2019). Optimal cultivation towards enhanced biomass and floridean starch production by *Porphyridium marinum*. Int. J. Biol. Macromol..

[12] Ben Amor F., Elleuch F., Ben Hlima H., Garnier M., Saint-Jean B., Barkallah M., Pichon C., Abdelkafi S., Fendri I. (2017). Proteomic analysis of the chlorophyta *Dunaliella* new strain AL-1 revealed global changes of metabolism during high carotenoid production. Mar. Drugs.

[13] Dammak M., Haase S.M., Miladi R., Ben Amor F., Barkallah M., Gosset D., Pichon C., Huchzermeyer B., Fendri I., Denis M. (2016). Enhanced lipid and biomass production by a newly isolated and identified marine microalga. Lipids Health Dis..

[14] Abdelkafi S., Chamkha M., Casalot L., Sayadi S., Labat M. (2005). Isolation and characterization of a novel *Bacillus* sp., strain YAS1, capable of transforming tyrosol under hypersaline conditions. FEMS Microbiol. Lett..

[15] Fendri I., Chaari A., Dhouib A., Jlassi B., Abousalham A., Carriere F., Sayadi S., Abdelkafi S. (2010). Isolation, identification and characterization of a new lipolytic *Pseudomonas* sp., strain AHD-1, from Tunisian soil. Environ. Technol..

[16] Dubois M., Gilles K.A., Hamilton J.K., Rebers P.T., Smith F. (1956). Colorimetric method for determination of sugars and related substances. Anal. Chem..

[17] Hentati F., Delattre C., Gardarin C., Desbrières J., Le Cerf D., Rihouey C., Michaud P., Abdelkafi S., Pierre G. (2020). Structural features and rheological properties of a sulphated xylogalactan-rich fraction isolated from Tunisian red seaweed *Jania ahaerens*. Appl. Sci..

[18] Hentati F., Delattre C., Ursu A.V., Desbrières J., Le Cerf D., Gardarin C., Abdelkafi S., Michaud P., Pierre G. (2018). Structural characterization and antioxidant activity of water-soluble polysaccharides from the Tunisian brown seaweed *Cystoseira compressa*. Carbohydr. Polym..

[19] Murashige T., Skoog F. (1962). A revised medium for rapid growth and bioassays with tobacco tissue cultures. Physiol. Planatrum.

[20] Velikova V., Yordanov I., Edreva A. (2000). Oxidative stress and some antioxidant systems in acid rain-treated bean plants protective role of exogenous polyamines. Plant Sci..

[21] Bradford M.M. (1976). A rapid and sensitive method for the quantitation of microgram quantities of protein utilizing the principle of protein-dye binding. Anal. Biochem..

[22] El-Shora H.M. (2002). Properties of phenylalanine ammonia-lyase from marrow cotyledons. Plant Sci..

[23] Livak K.J., Schmittgen T.D. (2001). Analysis of relative gene expression data using real-time quantitative PCR and the 2-ΔΔCT method. Methods.

[24] Ott F.D. (1967). A second record of *Porphyridium sordidum* Geitler 1. J. Phycol..

[25] Medina-Cabrera E.V., Rühmann B., Schmid J., Sieber V. (2020). Characterization and comparison of *Porphyridium Sordidum* and *Porphyridium Purpureum* concerning growth characteristics and polysaccharide production. Algal Res..

[26] Kroen W.K., Rayburn W.R. (1984). Influence of growth status and nutrients on extracellular polysaccharide synthesis by the soil alga *Chlamydomonas mexicana* (Chlorophyceae). J. Phycol..

[27] Díaz Bayona K.C., Garcés L.A. (2014). Effect of different media on exopolysaccharide and biomass production by the green microalga *Botryococcus Braunii*. J. Appl. Phycol..

[28] Patel A.K., Laroche C., Marcati A., Ursu A.V., Jubeau S., Marchal L., Petit E., Djelveh G., Michaud P. (2013). Separation and fractionation of exopolysaccharides from *Porphyridium cruentum*. Bioresour. Technol..

[29] Gaignard C., Gargouch N., Dubessay P., Delattre C., Pierre G., Laroche C., Fendri I., Abdelkafi S., Michaud P. (2019). New horizons in culture and valorization of red microalgae. Biotechnol. Adv..

[30] Han S.I.I., Jeon M.S., Heo Y.M., Kim S., Choi Y.E. (2020). Effect of *Pseudoalteromonas* sp. MEBiC 03485 on biomass production and sulfated polysaccharide biosynthesis in *Porphyridium cruentum* UTEX 161. Bioresour. Technol..

[31] Arad S., Levy-Ontman O. (2010). Red microalgal cell-wall polysaccharides: Biotechnological aspects. Curr. Opin. Biotechnol..

[32] Delattre C., Pierre G., Laroche C., Michaud P. (2016). Production, extraction and characterization of microalgal and cyanobacterial exopolysaccharides. Biotechnol. Adv..

[33] Arad S., Lerental Y.B., Dubinsky O. (1992). Effect of nitrate and sulfate starvation on polysaccharide formation in *Rhodella reticulata*. Bioresour. Technol..

[34] Michielse C.B., Rep M. (2009). Pathogen profile update: *Fusarium oxysporum*. Mol. Plant Pathol..

[35] Torres M.A., Jones J.D.G., Dangl J.L. (2006). Reactive oxygen species signaling in response to pathogens. Plant Physiol..

[36] Kong J.Q. (2015). Phenylalanine ammonia-lyase, a key component used for phenylpropanoids production by metabolic engineering. RSC Adv..

[37] Zheng F., Chen L., Zhang P., Zhou J., Lu X., Tian W. (2020). Carbohydrate polymers exhibit great potential as effective elicitors in organic agriculture: A review. Carbohydr. Polym..

[38] Barkallah M., Ben Atitallah A., Hentati F., Dammak M., Hadrich B., Fendri I., Ayadi M.-A., Michaud P., Abdelkafi S. (2019). Effect of *Spirulina platensis* biomass with high polysaccharides content on quality attributes of common carp (*Cyprinus carpio*) and common barbel (*Barbus barbus*) fish burgers. Appl. Sci..

[39] Cruz D., Vasconcelos V., Pierre G., Michaud P., Delattre C. (2020). Exopolysaccharides from cyanobacteria: Strategies for bioprocess development. Appl. Sci..

[40] Ozturk S., Aslim B. (2010). Modification of exopolysaccharide composition and production by three cyanobacterial isolates under salt stress. Environ. Sci. Pollut. Res..

[41] Jindal N., Singh D.P., Khattar J.I.S. (2011). Kinetics and physico-chemical characterization of exopolysaccharides produced by the cyanobacterium *Oscillatoria formosa*. World J. Microbiol. Biotechnol..

[42] Borah D., Nainamalai S., Gopalakrishnan S., Rout J., Alharbi N.S., Alharbi S.A., Nooruddin T. (2018). Biolubricant potential of exopolysaccharides from the cyanobacterium *Cyanothece epiphytica*. Appl. Microbiol. Biotechnol..

[43] Mishra A., Jha B. (2009). Isolation and characterization of extracellular polymeric substances from micro-algae *Dunaliella salina* under salt stress. Bioresour. Technol..

[44] Li T., Xu J., Wu H., Jiang P., Chen Z., Xiang W. (2019). Growth and biochemical composition of *Porphyridium purpureum* SCS-02 under different nitrogen concentrations. Mar. Drugs.

[45] Silva M.B.F., Azero E.G., Teixeira C.M.L.L., Andrade C.T. (2020). Influence of culture conditions on the production of Extracellular Polymeric Substances (EPS) by Arthrospira Platensis. Bioresour. Bioprocess..

[46] Cepák V., Přibyl P. (2018). Light intensity and nitrogen effectively control exopolysaccharide production by the green microalga botryococcus braunii (trebouxiophyceae). Genet. Plant Physiol..

[47] Aziz A., Poinssot B., Daire X., Adrian M., Bézier A., Lambert B., Joubert J.M., Pugin A. (2003). Laminarin elicits defense responses in grapevine and induces protection against *Botrytis cinerea* and *Plasmopara viticola*. Mol. Plant Microbe Interact..

[48] Dey P., Ramanujam R., Venkatesan G., Nagarathnam R. (2019). Sodium alginate potentiates antioxidant defense and PR proteins against early blight disease caused by *Alternaria solani* in *Solanum lycopersicum Linn*. PLoS ONE.

[49] Sangha J.S., Ravichandran S., Prithiviraj K., Critchley A.T., Prithiviraj B. (2010). Sulfated macroalgal polysaccharides λ-carrageenan and ι-carrageenan differentially alter *Arabidopsis thaliana* resistance to *Sclerotinia sclerotiorum*. Physiol. Mol. Plant Pathol..

[50] Ceron-Garcia A., Vargas-Arispuro I., Aispuro-Hernandez E., Angel M. (2012). Oligoglucan elicitor effects during plant oxidative stress. Cell Metab..

[51] Gunupuru L.R., Patel J.S., Sumarah M.W., Renaud J.B., Mantin E.G., Prithiviraj B. (2019). A plant biostimulant made from the marine brown algae Ascophyllum nodosum and chitosan reduce fusarium head blight and mycotoxin contamination in wheat. PLoS ONE.

[52] Jayaraj J., Wan A., Rahman M., Punja Z.K. (2008). Seaweed extract reduces foliar fungal diseases on carrot. Crop Prot..

[53] Rachidi F., Chanda M.J., Benhima R., El Mernissi N., Asfar A., Sbabou L., El Arroussi H. (2019). Effect of microalgae polysaccharides on biochemical and metabolomics pathways related to plant defense in *Solanum lycopersicum*. Appl. Biochem. Biotechnol..

[54] Trouvelot S., Héloir M.C., Poinssot B., Gauthier A., Paris F., Guillier C., Combier M., Trdá L., Daire X., Adrian M. (2014). Carbohydrates in plant immunity and plant protection: Roles and potential application as foliar sprays. Front. Plant Sci..

[55] Rachidi F., Benhima R., Kasmi Y., Sbabou L., Arroussi H.E. (2021). Evaluation of microalgae polysaccharides as biostimulants of tomato plant defense using metabolomics and biochemical approaches. Sci. Rep..

